# Effect of infliximab in sweet’s syndrome-pyoderma gangraenosum overlap syndrome

**DOI:** 10.1186/1546-0096-12-S1-P286

**Published:** 2014-09-17

**Authors:** Birgitte Mahler, Mia Glerup, Torben Steiniche, Troels Herlin

**Affiliations:** 1 Pediatrics, Randers regionshospital, Aarhus, Denmark; 2 Pediatrics, Aarhus University Hospital, Aarhus, Denmark; 3 Pathology, Aarhus University Hospital, Aarhus, Denmark

## Introduction

Both Sweet’s syndrome and pyoderma gangraenosum are neutrophilic dermatoses with abrupt onset of painful skin lesions. In Sweet’s syndrome systemic symptoms such as fever, headache and leucocytosis together with painful skin papules are often seen. Corticosteroid therapy is often sufficient treatment. Pyoderma gangraenosum is characterized by the development of painful ulcerative skin lesions and can be managed with anti-TNFα (infliximab).

## Objectives

We report a symptom overlap between these two conditions with an excellent response to infliximab.

## Methods

Case presentation.

## Results

A 7-month-old girl, first child of non-consanguineous Danish parents, was admitted with multiple painful inflammatory skin lesions. Pregnancy and delivery were without complications. The child was born at term with a birth weight of 3970 g and received her first vaccination (diphtheria, pertussis, tetanus, haemophilus influenzae and polio) 3 months old without any complication. Two weeks after she received the second vaccination 6 months old she developed a 3 x 4 cm large skin lesion consistent with pyoderma gangraenosum, at the injection site on her right thigh. The following month she developed numerous ½-1 cm large, round, necrotic, painful ulcerations on the extremities and the neck. CRP was elevated to 106 mg/L; haematology and immunoglobulins were normal. Biopsy showed lesions with diffuse, neutrophilic infiltrates but without granuloma or vasculitic abnormalities; the findings were therefore compatible with Sweet’s syndrome. On high-dose corticosteroid treatment with pulse methylprednisolone (10 mg/kg/day for 3 days) followed by oral prednisolone (2mg/kg/day) the initial response was marked. However, on repeated attempts of tapering off prednisolone she flared on even high doses of prednisolone (2 mg/kg/d) and azathioprine. She then received treatment with infliximab (7-8 mg/kg) resulting in a rapid and dramatic response within few days and went into remission and corticosteroids were finally tapered off. After 6 doses of infliximab the treatment was stopped. Remission has currently lasted one year after termination of medical treatment.

## Conclusion

This case shows that the clinicians have to be aware of the overlap between Sweet’s syndrome and pyoderma gangraenosum in order to identify patients that might benefit from anti-TNFα therapy.

## Disclosure of interest

None declared.

